# Investigation of bacterial microbiota variability in two allopatric populations of *Nyssomyia umbratilis*, susceptible and nonsusceptible to *Leishmania* (*Viannia) guyanensis* infection in the Amazon region

**DOI:** 10.1186/s13071-025-06976-9

**Published:** 2025-08-19

**Authors:** Eric Fabrício Marialva, Keillen Monick Martins-Campos, Victor Ramos de Almeida, Claudia María Ríos-Velasquez, Antônio Jorge Tempone, Felipe Arley Costa Pessoa, Yara Maria Traub-Cseko

**Affiliations:** 1 Programa de Pós-Graduação em Biologia da Interação Patógeno Hospedeiro – ILMD Fiocruz Amazônia, Manaus, AM Brazil; 2 Laborátório de Ecologia de Doenças Transmissíveis na Amazônia – ILMD Fiocruz Amazônia, Manaus, AM Brazil; 3 Laboratório de Biologia Molecular de Parasitos e Vetores – IOC Fiocruz, Rio de Janeiro, RJ Brazil

**Keywords:** Sand flies, Vector-borne, Metagenomics, *Leishmania*

## Abstract

**Background:**

Sand flies of the species *Nyssomyia umbratilis* (Diptera: Psychodidae: Phlebotominae) are vectors of the parasite *Leishmania* (*Viannia*) *guyanensis*, one of the etiological agents of cutaneous leishmaniasis in the Amazon region. In addition, *Ny. umbratilis* is a cryptic species, with populations showing differences in their ability to transmit the parasite. For instance, populations of *Ny. umbratilis* from the Manacapuru municipality (MAN), located on the south bank of the Negro river, in the Amazonas stated of Brazil, shows refractoriness to *Leishmania* infection, while populations from Rio Preto da Eva municipality (RPE), located on the north bank of the Negro river, are susceptible to infection. This lack of vectorial capacity may be caused by several factors, including the intestinal bacterial microbiota of sand flies.

**Methods:**

In this work, we carried out a metagenomic study of the intestinal microbiota of *Ny. umbratilis* populations from MAN and RPE. *Ny. umbratilis* females were collected in forested areas, sand fly midguts were dissected, DNA was extracted, and the 16 S rRNA gene sequenced to identify the bacterial composition of the microbiota.

**Results:**

In total, 16 phyla, 33 classes, 49 orders, 93 families, and 112 genera of bacteria were identified. The phylum Proteobacteria was the most frequent (85.9%) in both localities, followed by the phyla Bacteroidetes, Actinobacteria, and Firmicutes with, 9.9%, 4.9%, and 4.4%, respectively. In MAN, 84 genera were identified and 79 in RPE, with MAN having a greater richness compared with RPE. Among these, the genera *Rickettsia*, *Prevotella*, *Porphyromonas*, *Peptostreptococcus*, and *Caulobacter* were the most prevalent in MAN, and the genera *Rickettsia*, *Prevotella*, *Cryocola*, *Porphyromonas*, and *Caulobacter* were the most prevalent in RPE.

**Conclusions:**

Bacterial microbiota from MAN insects presents a greater diversity in relation to the RPE insects. Some of the identified bacteria have the potential to be used in alternative transmission control approaches as the development of transgenic vectors, and also, bacteria found exclusively in MAN sand flies may be candidates for a future transmission control approach to combat leishmaniasis in the Amazon region.

**Graphical Abstract:**

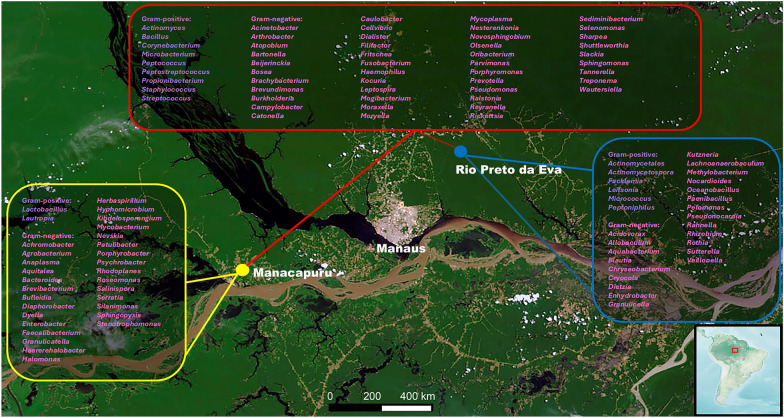

**Supplementary information:**

The online version contains supplementary material available at 10.1186/s13071-025-06976-9.

## Background

Leishmaniasis is among the six most prevalent endemic diseases transmitted by insects in the world; approximately 1 billion people are at risk of contracting leishmaniasis, and it is estimated that 30,000 new cases of visceral leishmaniasis and more than 1 million new cases of American cutaneous leishmaniasis (ACL) will occur annually [1].

Worldwide, 205,646 cases of ACL were reported in 2022, while in Brazil there were 12,878 cases, from which 8734 cases were registered in the Amazon region, with 809 of them in the state of Amazonas, AM. Seven species of *Leishmania* (Kinetoplastida: Trypanosomatidae) that cause cutaneous leishmaniasis in humans occur in the Amazon region: *Leishmania* (*Viannia*) *guyanensis*, *Leishmania* (*Leishmania*) *amazonensis*, *Leishmania* (*Viannia*) *braziliensis*, *Leishmania* (*Viannia*) *lainsoni*, *Leishmania* (*Viannia*) *shawi*, *Leishmania* (*Viannia*) *naiffi*, and *Leishmania* (*Viannia*) *lindenbergi*. These parasites are transmitted by different vector species [2]. *Nyssomyia umbratilis* (Diptera: Psychodidae) is the main vector of *Le. guyanensis*, the infectious agent of ACL in northern South America [3]. This species is widely distributed in the Amazon Basin, in Brazil, Bolivia, Colombia, French Guiana, Guyana, Peru, Suriname, and Venezuela [2, 4], and also has been registered in the Brazilian Atlantic Forest biome from the states of Pernambuco [5] and Alagoas [6], in the northeastern region of Brazil.

*Nyssomyia umbratilis* is a species complex with at least two incipient species [7–9]. The population of *Ny. umbratilis* from Rio Preto da Eva (RPE) municipality, Amazonas State, on the north bank of the Negro river, was found to be naturally infected with *Le. guyanensis*, while that from the Manacapuru (MAN) municipality, Amazonas State, on the south bank of the Negro river, was not infected, with the river apparently being a geographic barrier for gene flow between populations inhabiting opposite river banks [10]. Studies comparing the biology of these sand fly populations from the different sides of the Negro river, under laboratory conditions, showed that the population from RPE had greater survival and greater fecundity compared with females from MAN [11]. In addition, ex vivo assays showed a low rate of adhesion of *Le. guyanensis* to the intestines of *Ny. umbratilis* from MAN when compared with the intestines from RPE insects [12]. Experimental infection assays of *Ny. umbratilis*, fundamental to establishing the vectorial competence of the insects, have not yet been carried out owing to the difficulties in colonizing the species.

Differences in the insect susceptibility to pathogens could be related to many factors, such as immune responses, digestive enzymes, and the composition of the gut microbiota. Phlebotomine sand fly microbiota originates from the contact of the eggs with the female’s vaginal operculum and the environment where they are deposited. The larvae that emerge from these eggs feed on organic matter present in the environment, which also influences the formation of the microbiota [13]. When adult insects feed on sugary substances they can acquire still other organisms from the environment, and the sugars can influence the microbiota development [14–17]. Finally, when females take a blood meal, the microbiota may undergo further changes [18–22].

The microbiota has a direct relationship with the interaction between sand flies and *Leishmania*, and can influence this relationship positively [21, 23, 24] or negatively [25]. Moraes et al. [26, 27] demonstrated that the bacteria *Serratia marcescens* found in *Lutzomyia longipalpis* caused lysis in the cell wall of *Leishmania* (*Leishmania*) *infantum* and *Leishmania* (*Viannia*) *braziliensis* in in vitro experiments. When *Lu. longipalpis* was prefed with *Pseudozyma*, *Asaia*, or *Ochrobactrum*, and then infected with *Leishmania* (*Viannia*) *mexicana*, there was a reduced survival rate of the parasites. In contrast, when the sand flies were prefed with *Le. mexicana*, the parasite inhibited the development of the microorganisms in the insect [25]. A study with *Nyssomyia intermedia* demonstrated that the abundance and richness of the microbiota are modulated by the physiological condition of the sand fly [23]. The microbiota of *Lu. longipalpis* proved to be essential for the survival of *Leishmania*. Kelly et al. [24] observed that when antibiotics were administered to *Lu. longipalpis*, there was a decrease in the midgut microbiota, compromising the survival of *Le. infantum* within the vector and inhibiting the growth and differentiation of the parasite into the infective (metacyclic) form.

This work aimed to study the composition of the bacterial microbiota of two allopatric populations of *Ny. umbratilis* from the Amazon region, one susceptible and the other nonsusceptible to *Leishmania* infection. Studies about bacteria community and their interactions with the *Leishmania* and phlebotomine sand flies could be useful for developing control strategies on the basis of bacterial symbionts and paratransgenesis.

## Methods

### Ethics statement

The license to collect insects was obtained by SISBIO-ICMBio (Instituto Chico Mendes de Biodiversidade) no. 12186, and access to genetic patrimony obtained by SisGen (Sistema Nacional de Gestão do Patrimônio Genético e do Conhecimento Tradicional Associado) no. AFA0DE4, in the name of Felipe A.C. Pessoa.

### Phlebotomine sand fly field collection

**Fig 1.**
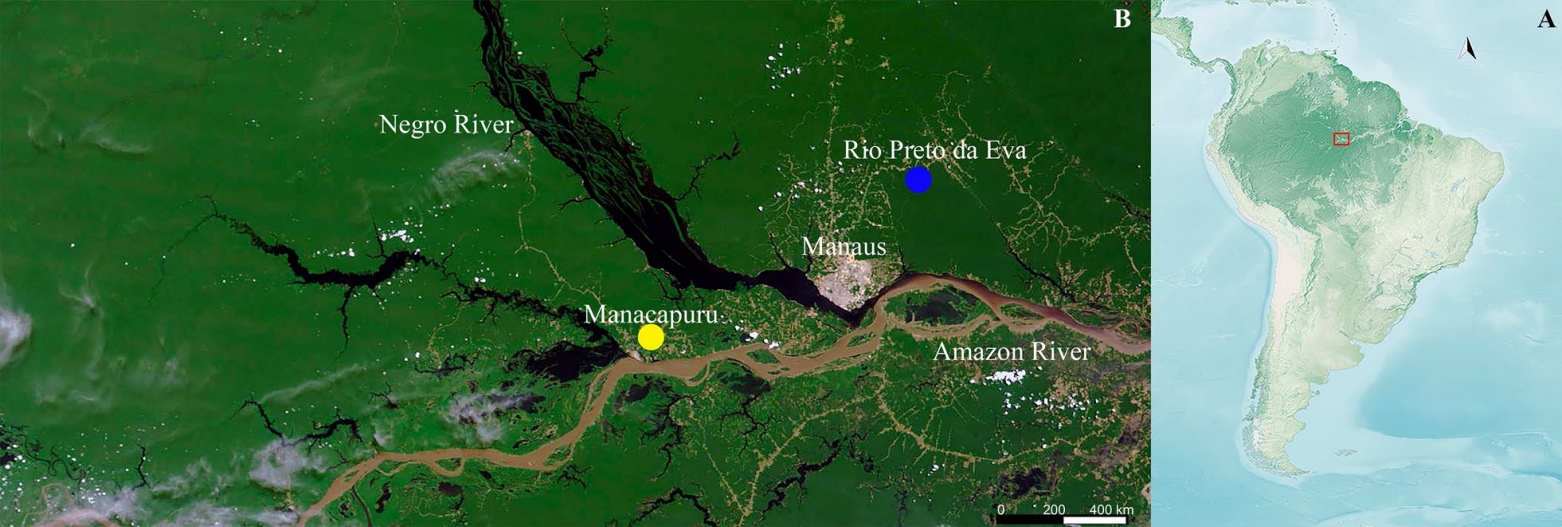
Sampling sites. (**A**) South America map detailing the field collection of phlebotomine sand fly sites in the Brazilian Amazon (red rectangle); (**B**) Manacapuru (yellow circle); Rio Preto da Eva (blue circle). Satellite photos: National Aeronautics and Space Administration

*Ny. umbratilis* were collected in RPE (north bank of Negro river, Amazonas State; 2°50′50″S/59°56′28″W) and MAN (south bank of Negro river, Amazonas State; 3°12′41″S/ 60°26′20’’W), approximately 150 km apart from each other (Fig. 1). Insects were collected manually by aspiration from crevices and tree trunks using modified Centers for Disease Control (CDC) light traps without a lamp, between 10:00 and 12:00 am.

Until dissection, the collected insects were kept in the insectary of the Laboratório de Ecologia de Doenças Transmissíveis na Amazônia at the Instituto Leônidas and Maria Deane—Fiocruz Amazônia, maintained at constant temperature and humidity (27 °C and 75% humidity). Taxonomic identification was carried out according to Young and Duncan [4] and Galati [28].

### Sample preparation

Nonengorged females were sterilized by submersion for 1 min in 1% sodium hypochlorite, 15–30 s in 70% ethanol, and then rinsed three times with phosphate buffered saline (PBS). Subsequently, the midguts were dissected in a drop of PBS using two entomological pins, and three pools of 50 midguts from each collection site were formed and stored in TRIzolTM Reagent (Thermo Fisher Scientific). The samples were processed in triplicates.

### DNA extraction and sequencing

The samples stored in TRIzolTM Reagent (Thermo Fisher Scientific) were sent to BPI Biotecnologia, Botucatu, São Paulo, which was responsible for DNA extraction and sequencing. Total DNA was extracted by the Trizol method according to the manufacturer’s protocol. Bacterial identification analysis was performed using the 16S region (16S rRNA gene). Polymerase chain reaction (PCR) of genomic DNA were performed using the 16S rRNA primers V3–V4: forward primer: (5ʹTCGTCGGCAGCGTCAGATGTGTATAAGAGACAGCCTACGGGNGGCWGCAG-3ʹ) and reverse primer (5ʹGTCTCGT GGGCTCGGAGATGTGTATAAGAGACAGGACTACHVGGGTATCTAATCC-3ʹ).

The reactions were performed in a final volume of 20 μL, containing 10 μL of Phusion High-Fidelity PCR Master Mix with HF Buffer (Thermo Fisher Scientific), 1 uL of 10 μM forward oligonucleotide, 1 uL of 10 μM reverse oligonucleotide, and 8 uL (~22 ng) of genomic DNA. The amplification program consisted of initial denaturation at 95 °C for 3 min, followed by 27 cycles of denaturation at 95 °C for 30 s, annealing at 55 °C for 30 s, extension at 72 °C for 30 s, and a final extension at 72 °C for 5 min. The amplification reactions were conducted in a Veriti^™^ Thermal Cycler (Applied Biosystems). After amplification reaction of each sample, amplification was verified by electrophoresis in a 2% agarose gel stained with UniSafe Dye 0.03% (v/v), ~600 bp (amplicon size).

### Indexing reaction

The indexers were then inserted into the common adapters, necessary for generating clusters and sequencing the samples. The indexing reaction was performed following the Nextera XT Index kit protocol (Illumina). Amplification reactions were carried out in a Veriti™ Thermal Cycler (Applied Biosystems).

The generated libraries were subjected to purification steps using Agencourt AMPure XP magnetic bead (Beckman Coulter), to remove very small fragments of the total population of molecules and primer residues. Thereafter, quantification was carried out using the Real Time PCR methodology using the KAPA-KK4824 Kit (Library Quantification Kit—Illumina/Universal) on the QuantStudio 3 equipment (Applied Biosystems), following the manufacturer’s protocol.

An equimolar pool of DNA was generated by normalizing all samples to 4 nM for sequencing, which was conducted using the Illumina MiSeq next generation sequencing system (Illumina^®^ Sequencing) and MiSeq Reagent Nano kit 500 cycles—2 × 250 bp reading. The program used for metagenomics analysis was QIIME2, version 2022.8 [29]. Raw sequence data were quality filtered using the q2-demux plugin, followed by final cleaning with DADA2 [30] (via q2-dada2). All amplicon sequence variants (ASVs) were aligned with mafft [31] (via q2-alignment) and used to construct a phylogenetic tree with fasttree2 [32] (via q2-phylogeny). Taxonomy was assigned to ASVs using the q2 feature classifier [33] and the Bayes naive classifier-sklearn taxonomy classifier against the GreenGenes reference sequences—138–99% for bacteria [34, 35].

### Microbiota data analysis

Data analyses were performed using Microsoft Excel software for data tabulation and analysis, GraphPad Prism for graphical representation and data analysis, Jvenn webtool graphical representation and Cytoscape (//www.cytoscape.org), a tool for visualizing complex networks between data, was used to visualize bacterial richness and shared bacteria in the two populations of *Ny. umbratilis*. The following analyses were carried out by BPI: the alpha and beta diversities of the sand flies’ bacterial communities were analyzed by QIIME2 [36, 37]. The group significance of alpha and beta diversity indices was calculated with QIIME2 plug-ins using the Kruskal–Wallis test and a permutational multivariate analysis of variance (PERMANOVA), respectively. A *P* value of 0.05 was considered significant. The Venn diagram was constructed using the Jvenn Webtool.

## Results

**Fig 2.**
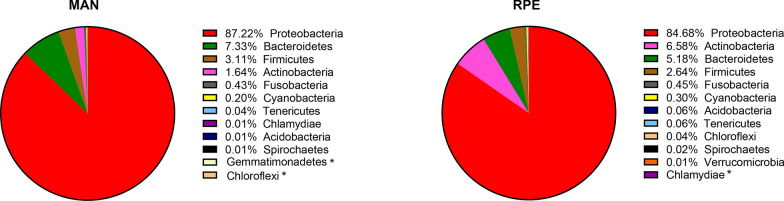
Distribution of bacteria at the phylum level in female guts of two *Nyssomyia umbratilis* populations from Manacapuru (MAN) and Rio Preto da Eva (RPE), *< 0.01%

**Fig 3.**
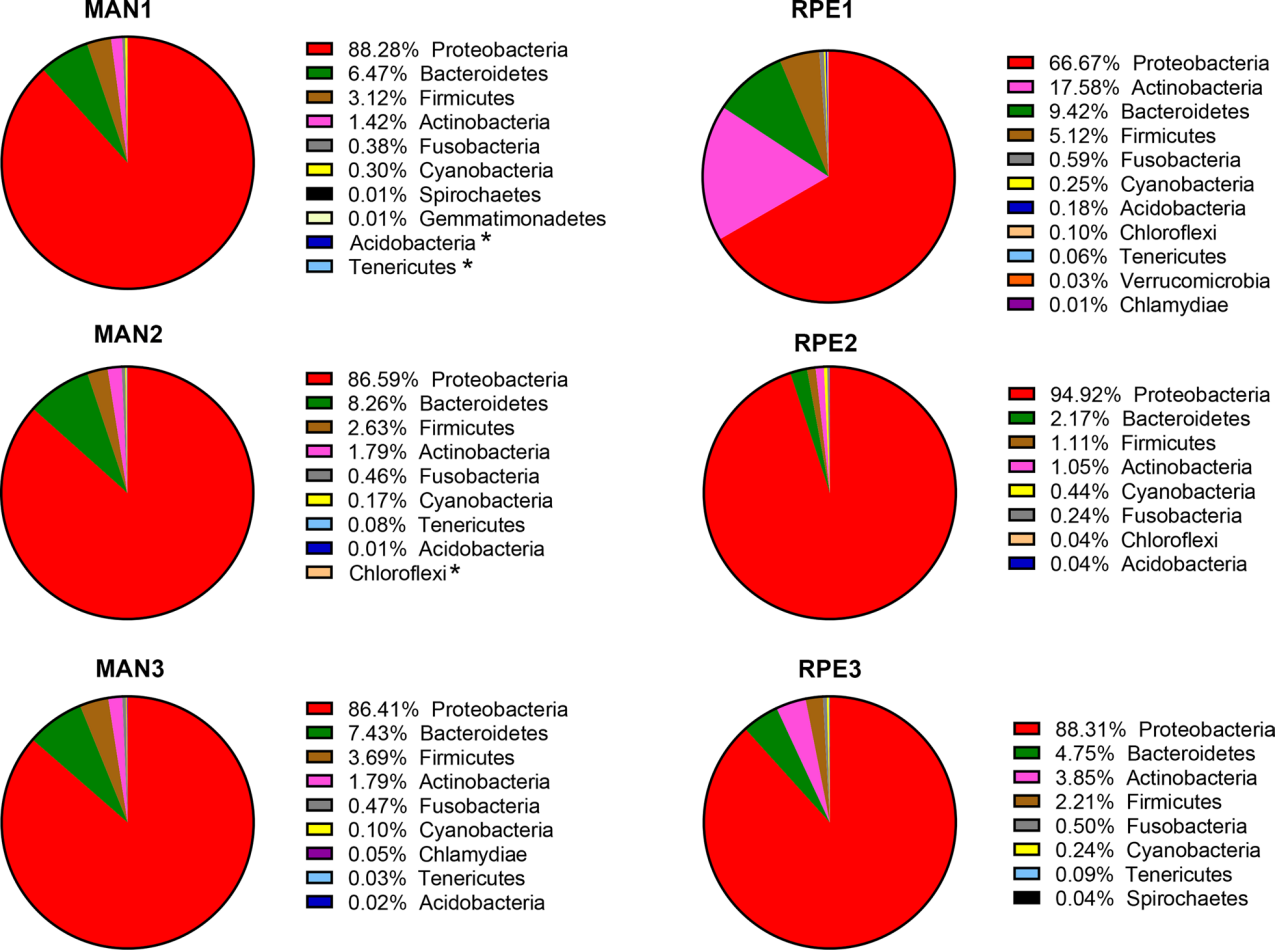
Abundance of bacteria at the phylum level in the female guts of two populations of *Nyssomyia umbratilis* in separate samples from the biological triplicate.* MAN* Manacapuru, *RPE* Rio Preto da Eva, *< 0.01%

The analysis of the 16 S rRNA gene from the bacterial microbiota of *Ny. umbratilis* from the populations of MAN and RPE showed high abundance and richness of the bacterial community, identifying 14 phyla, 33 classes, 46 orders, 93 families, and 112 genera. The phylum Proteobacteria had the highest number of readings in the intestines of females, with a representation of 87.22% and 84.68% of the total microbiota in MAN and RPE, respectively (Fig. 2). After Proteobacteria, the phyla Bacteroidetes, Actinobacteria, and Firmicutes were the most abundant in the sand flies, with 9.9%, 4.9%, and 4.4%, respectively. The relative abundance of bacterial phyla in different species and locations is summarized in Figs. 2 and 3. The alpha diversity index was higher in RPE compared with MAN, but there was no significant difference between the groups (*P* = 0.512); the PERMANOVA analysis of the beta diversity components also demonstrated no differences (*P* = 0.185).

**Fig 4.**
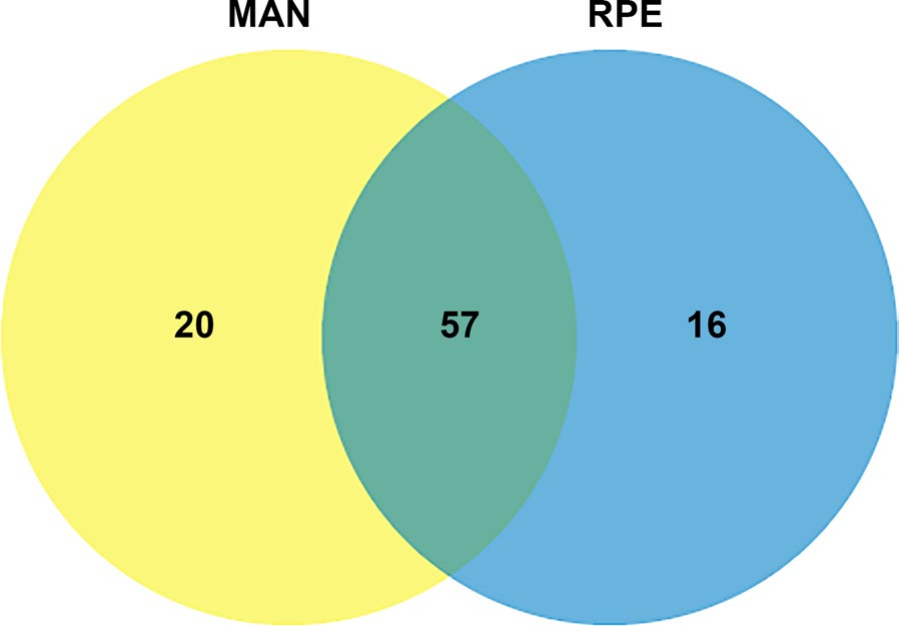
Venn diagram of the number of bacterial families present in the female gut of *Nyssomyia umbratilis* populations from Manacapuru (MAN) and Rio Preto da Eva (RPE), Amazonas State, Brazil

**Fig 5.**
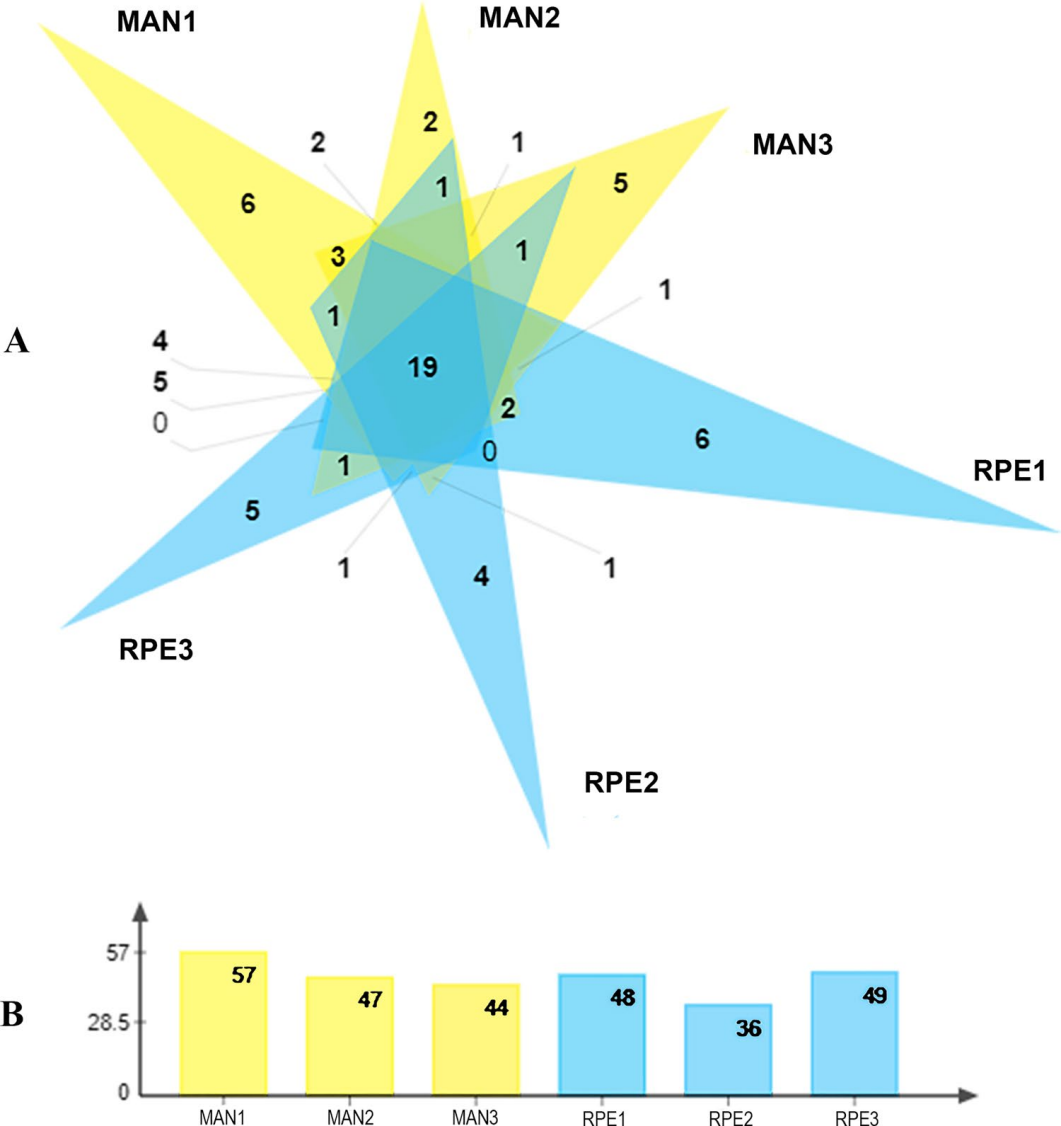
**A**: Venn diagram of the bacterial families present in the female gut of populations of *Nyssomyia umbratilis* from Manacapuru (MAN) and Rio Preto da Eva (RPE) in three different samples (1, 2, 3). **B**: Total number of bacterial families found in three different samples analyzed

A total of 93 families of bacterial taxa were detected in *Ny. umbratilis* midgut samples from the two locations (Fig. 4). The most abundant family was Rickettsiaceae with 84.6%, followed by Prevotellaceae (4.1%), Porphyromonadaceae (2.3%), and Microbacteriaceae (1.9%) (Table S1). An analysis of the Venn diagram revealed that 20 families were exclusive to MAN and 16 exclusive to RPE, while 57 families were shared (Fig. 4). When observed through sampling, a subset of 19 families of bacterial taxa was common in all six samplings (Fig. 5).

**Fig 6.**
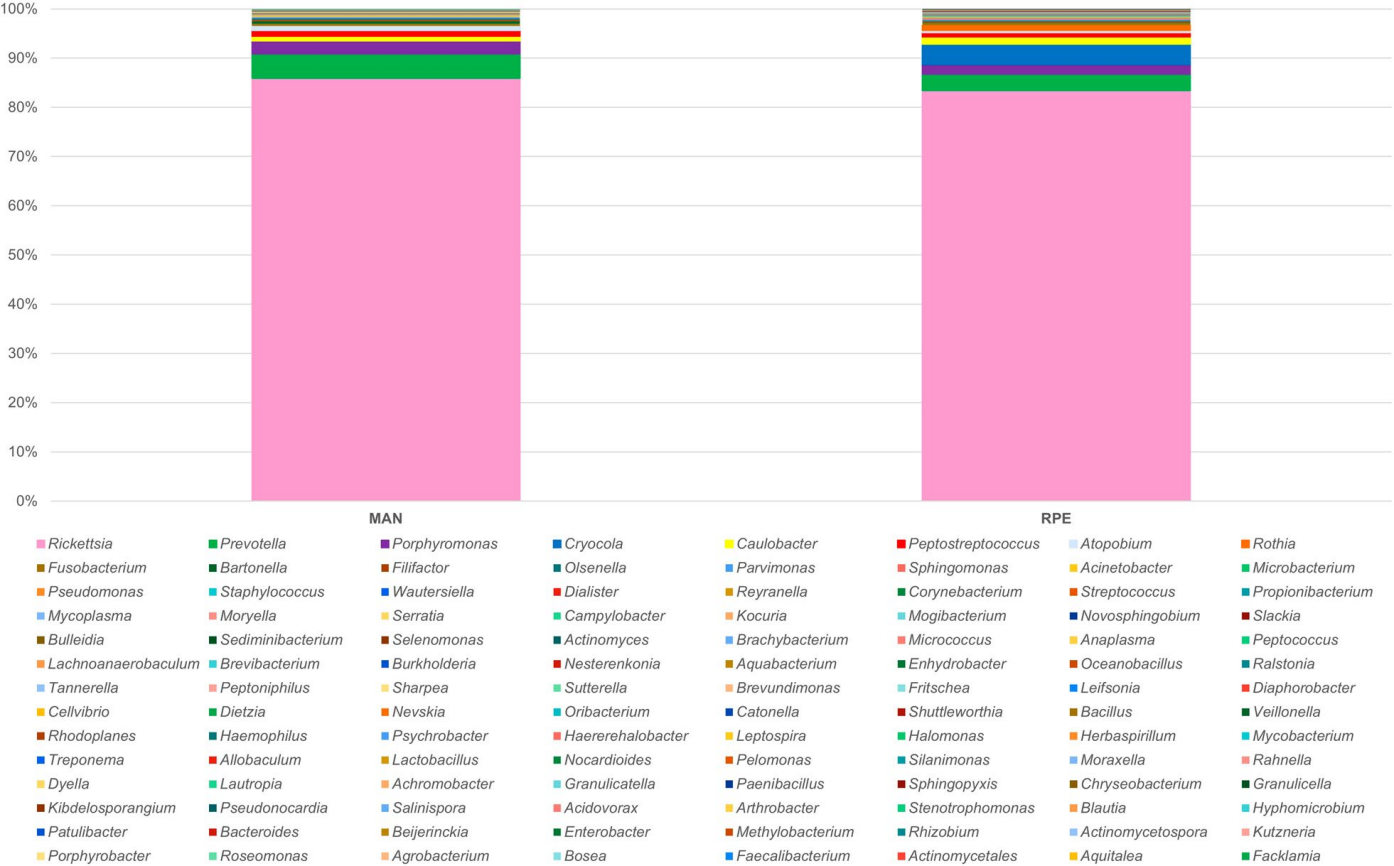
Abundance of bacterial genera identified in the gut microbiota of two populations of *Nyssomyia umbratilis* from Manacapuru (MAN) and Rio Preto da Eva (RPE), Amazonas State, Brazil

**Fig 7.**
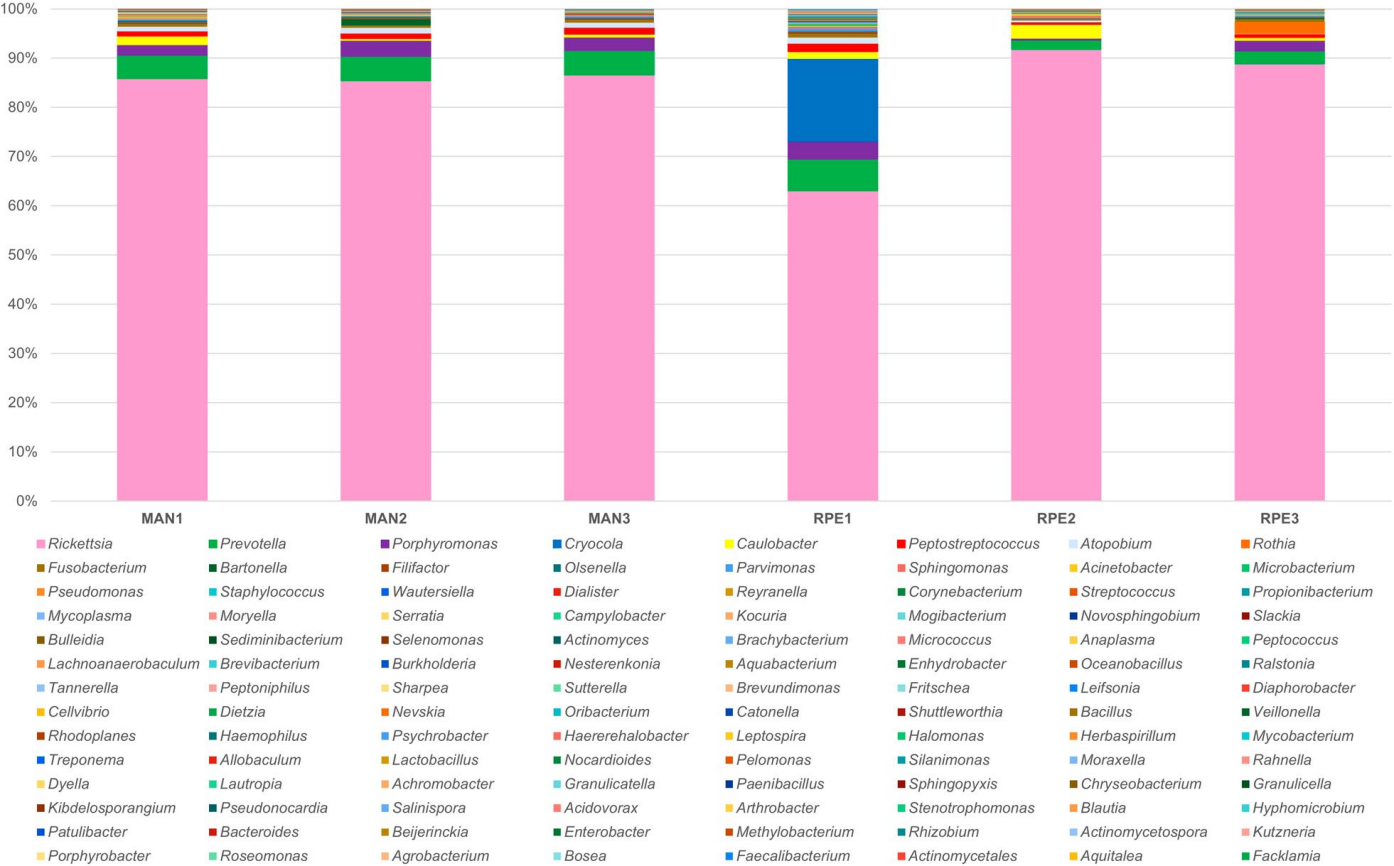
Abundance of bacterial genera identified in three different samples (1, 2, and 3) of gut microbiota of *Nyssomyia umbratilis* populations from Manacapuru (MAN) and Rio Preto da Eva (RPE), Amazonas State, Brazil

**Fig 8.**
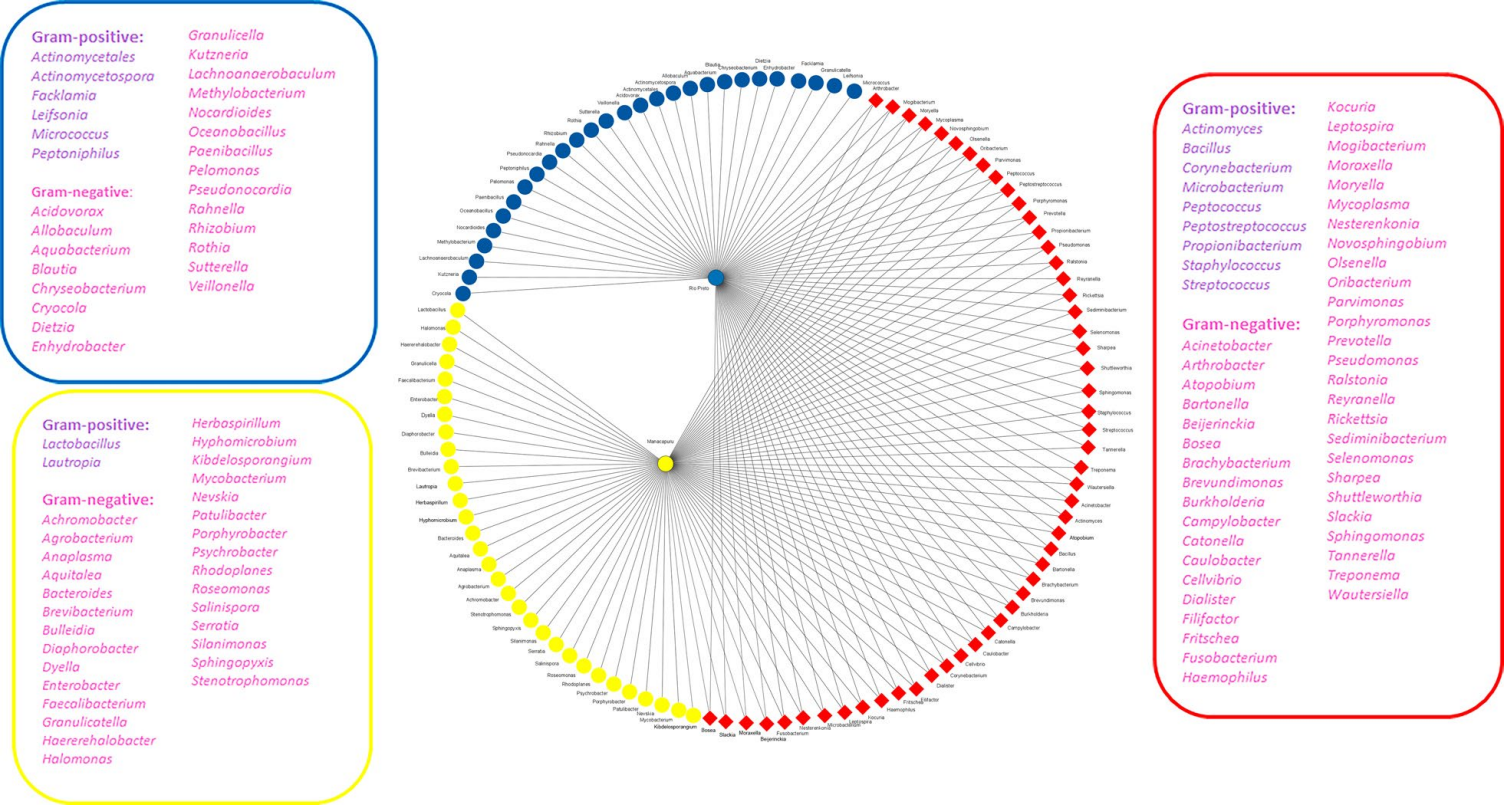
Network analysis showing shared (red diamond) and unique bacterial genera identified in the gut microbiota of *Nyssomyia umbratilis* populations from Manacapuru (Yellow circles and dots) and Rio Preto da Eva (Blue circles and dots)

A total of 112 bacterial genera were identified in the microbiota of *Ny. umbratilis* from MAN and RPE (Figs. 6 and 7), of which 46 bacterial genera were recorded for the first time in sand flies (Table S1). Furthermore, 95 genera consisted of Gram-negative bacteria, and 17 genera were Gram-positive (Fig. 8 and (Table S1). There were 84 genera identified in MAN and 79 in RPE, with MAN having a greater richness in relation to RPE (Figs. 6 and 7). Among these, five genera— *Rickettsia*, *Prevotella*, *Porphyromonas*, *Peptostreptococcus*, and *Caulobacter*—were the most prevalent in MAN, and five genera—*Rickettsia*, *Prevotella*, *Cryocola*, *Porphyromonas*, and *Caulobacter* —were the most prevalent in RPE (Figs. S1 and S2).

## Discussion

This study describes the composition of the microbiota of the midguts of two populations of Amazonian *Nyssomyia umbratilis*, apparently susceptible and nonsusceptible to *Leishmania* infection, and discusses possible implications for the differences found.

Our results show that the phylum Proteobacteria had an abundance of more than 84% in the RPE and MAN populations. This phylum colonizes several species of sand flies, and has also been found in greater abundance in the microbiota of other sand fly species, such as *Nyssomyia neivai* [38], *Nyssomyia intermedia* [23], *Lu. longipalpis* [21, 39], *Pintomyia evansi* [40, 41], and *Lutzomyia ayacuchensis* [42]. Proteobacteria are also present in several species of the genus *Phlebotomus*: *Phlebotomus duboscqi* [43], *Phlebotomus perniciosus* [44], *Phlebotomus papatasi* [39, 45], *Phlebotomus chinensis* [46], *Plebotomus kandelakii,*, *Phlebotomus halepensis*, and *Phlebotomus major* [47], demonstrating that this phylum has a high distribution in the microbiota of several species of sand flies around the world. The phylum Proteobacteria has also been found in high prevalence in mosquito species, e.g., in *Anopheles arabiensis* and *Anopheles funestus* in Tanzania [48], and in *Haemagogus leucocelaenus, Haemagogus capricornii*, *Aedes scapularis*, *Aedes serratus*, and *Psorophora ferox* in Brazil [49].

Our analyses also demonstrated that the phyla Bacteroidetes, Firmicutis, and Actinobacteria were the most representative after Proteobacteria, in both populations, with Bacteroidetes (7.33%), Firmicutis (3.11%), and Actinobacteria (1.64%) being more abundant in MAN population and Actinobacteria (6.58%), Bacteroidetes (5.18%), and Firmicutis (2.64%) being more abundant in RPE population. These phyla were also found in other sand fly species, such as *Ph. evansi* [40] and *Lu. ayacuchensis* [42], and were also the most representative phyla in *Phlebotomus kandelakii, Ph. halepensis,* and *Ph. major*. Proteobacteria, Bacteroidetes, Actinobacteria, and Firmicutes are the dominant phyla among bacterial communities in most insects, with Proteobacteria being the most abundant [50]. The wide range distribution of these four phyla could indicate that these bacteria have more affinity to invade and infect host insects, and they are probably common in the environments where these insects are found.

In the present study, the families Rickettsiaceae, Prevotellaceae, and Porphyromonadaceae were the three most abundant families in the RPE and MAN populations. These were found in the guts of unfed females. Studies carried out with *Lu. longipalpis* found Prevotellaceae and Porphyromonadaceae in unfed and gravid females [21], indicating that they are families of the normal microbiota of sand flies. Our study also demonstrated that the populations of *Ny. umbratilis* from MAN and RPE shared 19 families of bacteria. This diversity of families shared between populations of the same host species from different regions was also demonstrated in the work of Karimian et al. [47] with *Ph. kandelakii*, *Ph. halepensis,*, and *Ph. major*.

The genus *Rickettsia,* comprising intracellular bacteria, was the most abundant genus in both populations. Telleria et al. [13] pointed out that this species was associated with sand flies in areas of visceral leishmaniasis transmission. This does not seem the case here, since our work demonstrates the occurrence of this genus both in an endemic area of cutaneous leishmaniasis and an area where this disease does not occur, raising questions whether this bacterium really plays a role in the transmission of *Leishmania* in endemic areas.

Bacteria found only in refractory sand fly populations could play a role in parasite control. In this work, 31 bacteria genera were identified exclusively in the population refractory to infection (MAN) (Fig. 8). Some bacteria within these genera have shown antileishmanial effects or blocking transmission activity, such as *Serratia* and *Enterobacter*. *Serratia* has shown lytic effects against *Le. braziliensis* [27], *Le. infantum* [26], and *Le. mexicana* [25], and diminished significantly the survivorship of *Leishmania major* [51]. Campolina et al. [22] found that native bacteria isolated from *Lu. longipalpis* (*Bacillus*, *Enterobacter*, *Enterococcus*, *Erwinia*, *Escherichia*, *Staphylococcus* I, and *Pseudomonas*), when cocultivated with promastigotes of *Le. infantum*, *Le. major, Le. amazonensis*, and *Le. braziliensis*, caused death or reduction in the parasite numbers for all tested species. Furthermore, in the same research, experimental in vivo coinfection of *Lu. longipalpis* with *Le. infantum*, and *Serratia* caused a significant reduction of the number of parasites per sand fly. *Enterobacter cloacae* showed potential to limit the development of *Plasmodium* in *Anopheles* [52] and showed inhibition of *Le. infantum* promastigotes in vitro [53]; but it is beneficial to the *Le. major* development in *Ph. duboscqi* [54].

## Conclusions

The bacterial microbiota of *Ny. umbratilis* from the MAN and RPE populations showed high abundance and richness, with MAN bacterial microbiota having a greater diversity in relation to the RPE population. Some of the bacterial genera found in the MAN population could be possible candidates for paratransgenic approaches to reduce *Leishmania* transmission in the Amazon region. Considering the promising results mentioned above of the inhibition of malaria parasites by specific bacteria, we intend to focus on the microbiota of MAN, with the intention of identifying bacteria potentially responsible for this refractoriness.

## Supplementary information

Additional file 1.

## Data Availability

Data are provided within the manuscript or supplementary information files. Sequence data that support the findings of this study have been deposited in NCBI with accession code SUB1543923.

## References

[1] Word Health Organization. Leishmaniasis. 2024. Available from: https://www.who.int/news-room/fact-sheets/detail/leishmaniasis

[2] Rangel EF, Shaw JJ. Brazilian Sand Flies: Biology, taxonomy, medical importance and control. Cham: Springer; 2018. p. 341–80.

[3] Ready PD, Arias JR, Freitas RA. A pilot study to control *Lutzomyia umbratilis* (Diptera: Psychodidae), the major vector of *Leishmania braziliensis guyanensis*, in a peri-urban rainforest of Manaus, Amazonas State, Brazil. Mem Inst Oswaldo Cruz. 1985;80:27–36. 4088044 10.1590/s0074-02761985000100005

[4] Young DG, Duran MA. Guide to the identification and geographic distribution of *Lutzomyia* sand flies in Mexico, the West Indies, Central and South America (Diptera:Psychodidae). American Entomology Institue, Gainesville, Florida, 1944;419

[5] Balbino VQ, Marcondes CB, Alexander B, Luna LK, Lucena MM, Mendes AC, et al. First report of *Lutzomyia* (*Nyssomyia*) *umbratilis* Ward & Frahia, 1977 outside of Amazonian Region, in Recife, State of Pernambuco, Brazil (Diptera: Psychodidae: Phlebotominae). Mem Inst Oswaldo Cruz. 2001;96:315–7. 11313636 10.1590/s0074-02762001000300005

[6] de Souza Freitas MT, dos Santos CFR, de Andrade EM, Marcondes CB, de Queiroz BV, Pessoa AC. New records of phlebotomine sand flies (Diptera: Psychodidae) from the state of Alagoas, northeast of Brazil. J Med Entomol. 2018;55:242–7. 29029319 10.1093/jme/tjx175

[7] Scarpassa VM, Alencar RB. *Lutzomyia umbratilis*, the main vector of *Leishmania guyanensis*, represents a novel species complex? PLoS ONE. 2012;7:e37341. 22662146 10.1371/journal.pone.0037341 PMC3356248

[8] de Souza Freitas MT, Ríos-Velasquez CM, Costa CRL, Figueirêdo CAS, Aragão NC, da Silva LG, et al. Phenotypic and genotypic variations among three allopatric populations of *Lutzomyia umbratilis*, main vector of *Leishmania guyanensis*. Parasites Vectors. 2015;8:448. 26338469 10.1186/s13071-015-1051-7 PMC4559179

[9] de Souza Freitas MT, Ríos-Velasquez CM, da Silva LG, Lima Costa CR, Marcelino A, Leal-Balbino TC, et al. Analysis of the genetic structure of allopatric populations of *Lutzomyia umbratilis* using the period clock gene. Acta Trop. 2016;154:149–54. 26655040 10.1016/j.actatropica.2015.11.014

[10] Arias JR, de Freitas RA. Sobre os vetores de leishmaniose cutânea na Amazônia central do Brasil. 2: incidência de flagelados em flebótomos selváticos. Acta Amaz. 1978;8:387–96.

[11] Justiniano SCB, Chagas AC, Pessoa FAC, Queiroz RG. Comparative biology of two populations of *Lutzomyia umbratilis* (Diptera: Psychodidae) of Central Amazonia, Brazil, under laboratory conditions. Braz J Biol. 2004;64:227–35. 15462295 10.1590/s1519-69842004000200007

[12] Soares RP, Nogueira PM, Secundino NF, Marialva EF, Ríos-Velásquez CM, Pessoa FAC. *Lutzomyia umbratilis* from an area south of the Negro River is refractory to in vitro interaction with *Leishmania guyanensis*. Mem Inst Oswaldo Cruz. 2018;113:202–5. 29412360 10.1590/0074-02760170425 PMC5804313

[13] Telleria EL, Martins-da-Silva A, Tempone AJ, Traub-Csekö YM. *Leishmania*, microbiota and sand fly immunity. Parasitology. 2018;145:1336–53. 29921334 10.1017/S0031182018001014 PMC6137379

[14] Wallbanks KR, Moore JS, Bennett LR, Soren R, Molyneux DH, Carlin JM, et al. Aphid derived sugars in the neotropical sandfly *Lutzomyia peruensis*. Trop Med Parasitol. 1991;42:60–2. 2052859

[15] Añez N, Lugo A, Loaiza A, Nieves E, Orozco J. Sugars in the alimentary canal of *Lutzomyia youngi* (Diptera: Phlebotominae). Med Vet Entomol. 1994;8:38–42. 8161842 10.1111/j.1365-2915.1994.tb00381.x

[16] Cameron MM, Pessoa FAC, Vasconcelos AW, Ward RD. Sugar meal sources for the phlebotomine sandfly *Lutzomyia longipalpis* in Ceará State, Brazil. Med Vet Entomol. 1995;9:263–72. 7548943 10.1111/j.1365-2915.1995.tb00132.x

[17] Muller G, Schlein Y. Nectar and honeydew feeding of *Phlebotomus papatasi* in a focus of *Leishmania major* in Neot Hakikar oasis. J Vector Ecol. 2004;29:154–8. 15266752

[18] de Oliveira SMP, de Moraes BA, Gonçalves CA, Giordano-Dias CM, d’Almeida JM, Asensi MD, et al. Prevalência da microbiota no trato digestivo de fêmeas de *Lutzomyia longipalpis* (Lutz & Neiva, 1912) (Diptera: Psychodidae) provenientes do campo. Rev Soc Bras Med Trop. 2000;33:319–22. 10967602 10.1590/s0037-86822000000300012

[19] de Oliveira SMP, de Morais BA, Gonçalves CA, Giordano-Dias CM, Vilela ML, Brazil RP, et al. Microbiota do trato digestivo de fêmeas de *Lutzomyia longipalpis* (Lutz & Neiva, 1912) (Diptera: Psychodidae) provenientes de colônia alimentadas com sangue e com sangue e sacarose. Cad Saúde Pública. 2001;17:229–32. 11241946

[20] Gouveia C, Asensi MD, Zahner V, Rangel EF, de Oliveira SMP. Study on the bacterial midgut microbiota associated to different Brazilian populations of *Lutzomyia longipalpis* (Lutz & Neiva) (Diptera: Psychodidae). Neotrop Entomol. 2008;37:597–601. 19061048 10.1590/s1519-566x2008000500016

[21] Pires ACAM, Villegas LEM, Campolina TB, Orfanó AS, Pimenta PFP, Secundino NFC. Bacterial diversity of wild-caught *Lutzomyia longipalpis* (a vector of zoonotic visceral leishmaniasis in Brazil) under distinct physiological conditions by metagenomics analysis. Parasit Vectors. 2017;10:627. 29284535 10.1186/s13071-017-2593-7 PMC5747039

[22] Campolina TB, Villegas LEM, Monteiro CC, Pimenta PFP, Secundino NFC. Tripartite interactions: *Leishmania*, microbiota and *Lutzomyia longipalpis*. PLoS Negl Trop Dis. 2020;14:e0008666. 33052941 10.1371/journal.pntd.0008666 PMC7556539

[23] Monteiro CC, Villegas LEM, Campolina TB, Pires ACMA, Miranda JC, Pimenta PFP, et al. Bacterial diversity of the American sand fly *Lutzomyia intermedia* using high-throughput metagenomic sequencing. Parasit Vectors. 2016;9:480. 27581188 10.1186/s13071-016-1767-z PMC5007851

[24] Kelly PH, Bahr SM, Serafim TD, Ajami NJ, Petrosino JF, Meneses C, et al. The gut microbiome of the vector *Lutzomyia longipalpis* is essential for survival of *Leishmania infantum*. ASM Journals. 2017;8:10–128. 10.1128/mBio.01121-16 PMC5241394 28096483

[25] SantAnna MR, Diaz-Albiter H, Aguiar-Martins K, Al Salem WS, Cavalcante RR, Dillon VM, et al. Colonisation resistance in the sand fly gut: *Leishmania* protects *Lutzomyia longipalpis* from bacterial infection. Parasit Vectors. 2014;7:329. 25051919 10.1186/1756-3305-7-329 PMC4112039

[26] Moraes CS, Seabra SH, Castro DP, Brazil RP, de Souza W, Garcia ES, et al. *Leishmania* (*Leishmania*) *chagasi* interactions with *Serratia marcescens*: ultrastructural studies, lysis and carbohydrate effects. Exp Parasitol. 2008;118:561–8. 18206142 10.1016/j.exppara.2007.11.015

[27] Moraes CS, Seabra SH, Albuquerque-Cunha JM, Castro DP, Genta FA, Souza W, et al. Prodigiosin is not a determinant factor in lysis of *Leishmania* (*Viannia*) *braziliensis* after interaction with *Serratia marcescens* d-mannose sensitive fimbriae. Exp Parasitol. 2009;122:84–90. 19303010 10.1016/j.exppara.2009.03.004

[28] Galati E. Morfologia e terminologia de Phlebotominae (Diptera: Psychodidae). Classificação e identificação de táxons das Américass. Vol I. Apostila da Disciplina Bioecologia e Identificação de Phlebotominae do Programa de Pós-Graduação em Saúde Pública. Faculdade de Saúde Pública da Universidade de São Paulo, São Paulo.133p. 2021. Available from: http://www.fsp.usp.br/egalati.

[29] Bolyen E, Rideout JR, Dillon MR, Bokulich NA, Abnet CC, Al-Ghalith GA, et al. Reproducible, interactive, scalable and extensible microbiome data science using QIIME 2. Nat Biotechnol. 2019;37:852–7. 31341288 10.1038/s41587-019-0209-9 PMC7015180

[30] Callahan BJ, McMurdie PJ, Rosen MJ, Han AW, Johnson AJA, Holmes SP. DADA2: high-resolution sample inference from Illumina amplicon data. Nat Methods. 2016;13:581–3. 27214047 10.1038/nmeth.3869 PMC4927377

[31] Katoh K, Misawa K, Kuma K, Miyata T. MAFFT: a novel method for rapid multiple sequence alignment based on fast Fourier transform. Nucleic Acids Res. 2002;30:3059–66. 12136088 10.1093/nar/gkf436 PMC135756

[32] Price MN, Dehal PS, Arkin AP. FastTree 2—Approximately maximum-likelihood trees for large alignments. PLoS ONE. 2010;5:e9490. 20224823 10.1371/journal.pone.0009490 PMC2835736

[33] Bokulich NA, Dillon MR, Zhang Y, Rideout JR, Bolyen E, Li H, et al. q2-longitudinal: longitudinal and paired-sample analyses of microbiome data. mSystems. 2018;3:e00219-18. 10.1128/msystems.00219-18. 30505944 10.1128/mSystems.00219-18 PMC6247016

[34] Quast C, Pruesse E, Yilmaz P, Gerken J, Schweer T, Yarza P, et al. The SILVA ribosomal RNA gene database project: improved data processing and web-based tools. Nucleic Acids Res. 2012;41:D590–6. 23193283 10.1093/nar/gks1219 PMC3531112

[35] Yilmaz B, Portugal S, Tran TM, Gozzelino R, Ramos S, Gomes J, et al. Gut microbiota elicits a protective immune response against malaria transmission. Cell. 2014;159:1277–89. 25480293 10.1016/j.cell.2014.10.053 PMC4261137

[36] Lozupone C, Knight R. UniFrac: a new phylogenetic method for comparing microbial communities. Appl Environ Microbiol. 2005;71:8228–35. 16332807 10.1128/AEM.71.12.8228-8235.2005 PMC1317376

[37] Lozupone CA, Hamady M, Kelley ST, Knight R. Quantitative and qualitative β diversity measures lead to different insights into factors that structure microbial communities. Appl Environ Microbiol. 2007;73:1576–85. 17220268 10.1128/AEM.01996-06 PMC1828774

[38] Machado VE, Martins PMM, Ferreira H, Ferro M, Bacci M, Pinto MC. Bacterial groups associated with *Nyssomyia neivai* (Diptera: Psychodidae) sandflies. J Vector Borne Dis. 2014;51:137–9. 24947222

[39] Tabbabi A, Mizushima D, Yamamoto DS, Kato H. Effects of host species on microbiota composition in *Phlebotomus* and *Lutzomyia* sand flies. Parasit Vectors. 2023;16:310. 37653518 10.1186/s13071-023-05939-2 PMC10472604

[40] Vivero RJ, Jaramillo NG, Cadavid-Restrepo G, Soto SIU, Herrera CXM. Structural differences in gut bacteria communities in developmental stages of natural populations of *Lutzomyia evansi* from Colombia’s Caribbean coast. Parasit Vectors. 2016;9:496. 27618991 10.1186/s13071-016-1766-0 PMC5020466

[41] Vivero RJ, Villegas-Plazas M, Cadavid-Restrepo GE, Herrera CXM, Uribe SI, Junca H. Wild specimens of sand fly phlebotomine *Lutzomyia evansi*, vector of leishmaniasis, show high abundance of *Methylobacterium* and natural carriage of *Wolbachia* and *Cardinium* types in the midgut microbiome. Sci Rep. 2019;9:17746. 31780680 10.1038/s41598-019-53769-z PMC6883041

[42] Tabbabi A, Watanabe S, Mizushima D, Caceres AG, Gomez EA, Yamamoto DS, et al. Comparative analysis of bacterial communities in *Lutzomyia ayacuchensis* populations with different vector competence to *Leishmania* parasites in Ecuador and Peru. Microorganisms. 2020;9:68. 33383851 10.3390/microorganisms9010068 PMC7823435

[43] Volf P, Kiewegová A, Nemec A. Bacterial colonisation in the gut of *Phlebotomus duboscqi* (Diptera: Psychodidae): transtadial passage and the role of female diet. Folia Parasitol. 2002;49:73–7. 10.14411/fp.2002.014 11993554

[44] Fraihi W, Fares W, Perrin P, Dorkeld F, Sereno D, Barhoumi W, et al. An integrated overview of the midgut bacterial flora composition of *Phlebotomus perniciosus*, a vector of zoonotic visceral leishmaniasis in the Western Mediterranean Basin. PLoS Negl Trop Dis. 2017;11:e0005484. 28355207 10.1371/journal.pntd.0005484 PMC5386300

[45] Maleki-Ravasan N, Oshaghi MA, Afshar D, Arandian MH, Hajikhani S, Akhavan AA, et al. Aerobic bacterial flora of biotic and abiotic compartments of a hyperendemic zoonotic cutaneous leishmaniasis (ZCL) focus. Parasit Vectors. 2015;8:63. 25630498 10.1186/s13071-014-0517-3 PMC4329651

[46] Li K, Chen H, Jiang J, Li X, Xu J, Ma Y. Diversity of bacteriome associated with *Phlebotomus chinensis* (Diptera: Psychodidae) sand flies in two wild populations from China. Sci Rep. 2016;6:36406. 27819272 10.1038/srep36406 PMC5098245

[47] Karimian F, Koosha M, Choubdar N, Oshaghi MA. Comparative analysis of the gut microbiota of sand fly vectors of zoonotic visceral leishmaniasis (ZVL) in Iran; host-environment interplay shapes diversity. PLoS Negl Trop Dis. 2022;16:e0010609. 35853080 10.1371/journal.pntd.0010609 PMC9337680

[48] Baldini F, Rougé J, Kreppel K, Mkandawile G, Mapua SA, Sikulu-Lord M, et al. First report of natural *Wolbachia* infection in the malaria mosquito *Anopheles arabiensis* in Tanzania. Parasit Vectors. 2018;11:635. 30545384 10.1186/s13071-018-3249-y PMC6293665

[49] Da Silva H, Oliveira TMP, Sallum MAM. Bacterial community diversity and bacterial interaction network in eight mosquito species. Genes. 2022;13:2052. 36360289 10.3390/genes13112052 PMC9690548

[50] Jones RT, Sanchez LG, Fierer N. A cross-taxon analysis of insect-associated bacterial diversity. PLoS ONE. 2013;8:e61218. 23613815 10.1371/journal.pone.0061218 PMC3628706

[51] Hassan MI, Al-Sawaf BM, Fouda MA, Al-Hosry S, Hammad KM. A recent evaluation of the sandfly, *Phlepotomus papatas*i midgut symbiotic bacteria effect on the survivorship of *Leshmania major*. J Anc Dis Prev Rem. 2014;2:110.

[52] Dehghan H, Oshaghi MA, Moosa-Kazemi SH, Yakhchali B, Vatandoost H, Maleki-Ravasan N, et al. Dynamics of transgenic enterobacter cloacae expressing green fluorescent protein defensin (GFP-D) in *Anopheles stephensi* under laboratory condition. J Arthropod Borne Dis. 2017;11:515–32. 29367928 PMC5775158

[53] Vivero Gómez RJ, Cadavid Restrepo GE, Moreno Herrera CX, Ospina V, Uribe SI, Robledo SM. Antagonistic effect of bacteria isolated from the digestive tract of *Lutzomyia evansi* against promastigotes of *Leishmania infantum*, antimicrobial activities and susceptibility to antibiotics. AIDS Patient Care STDs. 2016;06:760–75.

[54] Louradour I, Monteiro CC, Inbar E, Ghosh K, Merkhofer R, Lawyer P, et al. The midgut microbiota plays an essential role in sand fly vector competence for *Leishmania major*. Cell Microbiol. 2017;19:e12755. 10.1111/cmi.12755 PMC5587349 28580630

