# Correction: Investigation of bacterial microbiota variability in two allopatric populations of *Nyssomyia umbratilis*, susceptible and nonsusceptible to *Leishmania (Viannia) guyanensis * infection in the Amazon region

**DOI:** 10.1186/s13071-025-07034-0

**Published:** 2025-09-29

**Authors:** Eric Fabrício Marialva, Keillen Monick Martins-Campos, Victor Ramos de Almeida, Claudia María Ríos-Velasquez, Antônio Jorge Tempone, Felipe Arley Costa Pessoa, Yara Maria Traub-Cseko

**Affiliations:** 1 Programa de Pós-Graduação em Biologia da Interação Patógeno Hospedeiro – ILMD Fiocruz Amazônia, Manaus, AM Brazil; 2 Laborátório de Ecologia de Doenças Transmissíveis na Amazônia – ILMD Fiocruz Amazônia, Manaus, AM Brazil; 3 Laboratório de Biologia Molecular de Parasitos e Vetores – IOC Fiocruz, Rio de Janeiro, RJ Brazil

**Correction: Parasites & Vectors (2025) 18:354** 10.1186/s13071-025-06976-9

Following publication of the original article [1], it came to the authors’ attention that ‘Victor’ had been incorrectly spelled as ‘Victo’ in the third author’s name. The author list has since been corrected. The authors thank you for reading this erratum and apologize for any inconvenience caused.
