# Supplementary material for: Investigation of bacterial microbiota variability in two allopatric populations of Nyssomyia umbratilis, susceptible and nonsusceptible to Leishmania (Viannia) guyanensis infection in the Amazon region
Source: Parasit Vectors. 2025 Aug 19;18:354. doi: 10.1186/s13071-025-06976-9 (PMC12366322; doi:10.1186/s13071-025-06976-9)
Supplement: Supplementary file 1 — Additional file 1. [file 13071_2025_6976_MOESM1_ESM.docx]

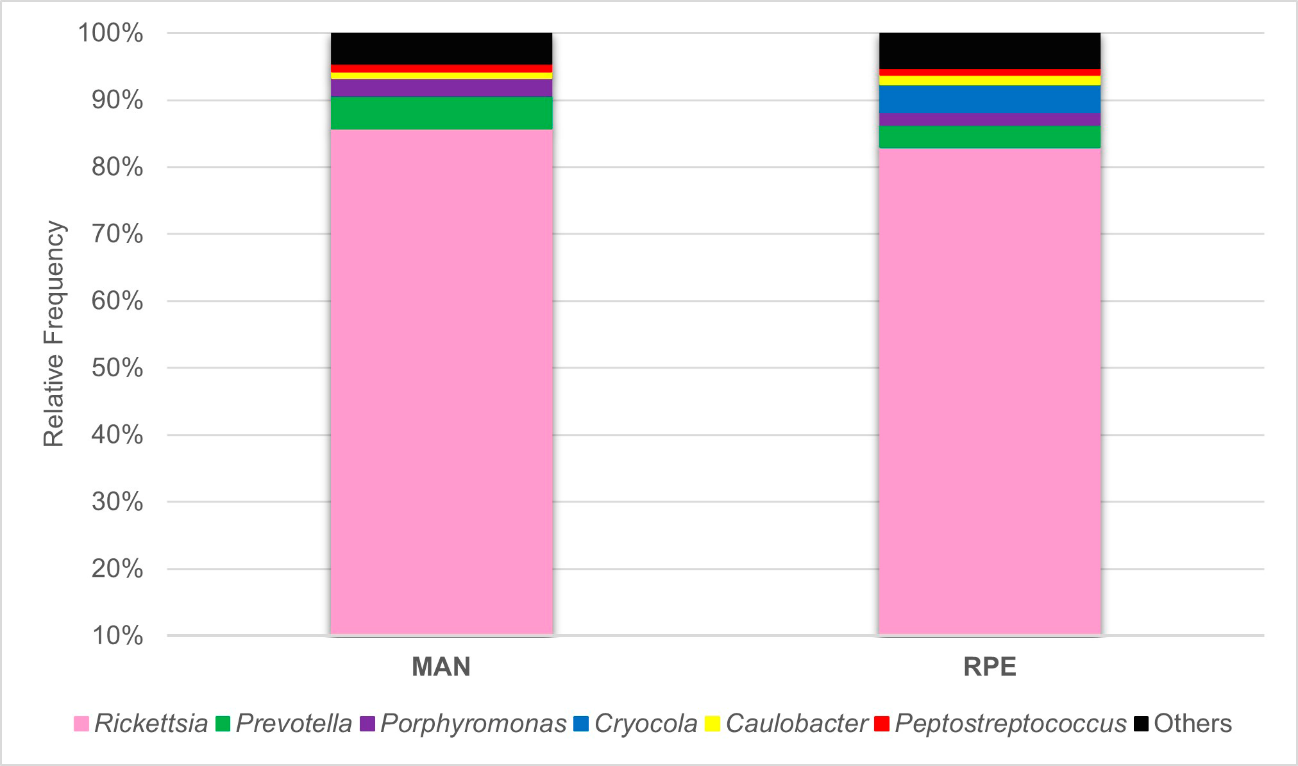


Figure S1. Relative frequency of the most abundant bacterial genera identified in the gut microbiota of two of *Nyssomyia umbratilis* populations from Manacapuru (MAN)and Rio Preto da Eva (RPE), Amazonas State, Brazil


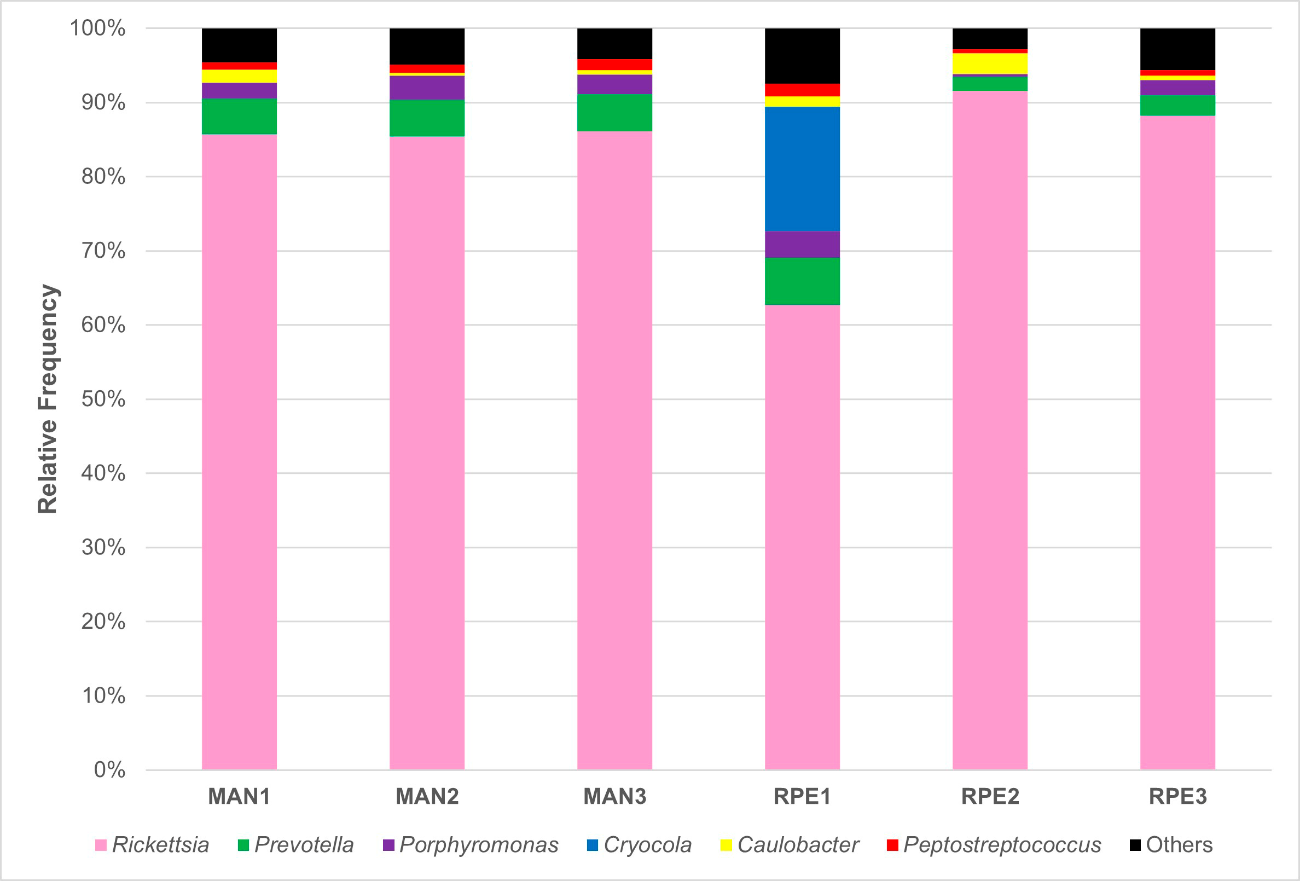


Figure S2. Relative frequency of the most abundant bacterial genera presents in three different samples (1, 2 and 3) in the gut microbiota of two *Nyssomyia umbratilis* populations from: Manacapuru (MAN) and Rio Preto da Eva (RPE), Amazonas State, Brazil.

Table S1: Taxonomic classification of OTUs from metagenomics of *Nyssomyia* *umbratilis* populations. MAN: Manacapuru; RPE: Rio Preto da Eva.

| Family | Genus | Gram +/- | No. of OTUs in | | | | | | |
| --- | --- | --- | --- | --- | --- | --- | --- | --- | --- |
|  |  |  | MAN1 | MAN2 | MAN3 | RPE1 | RPE2 | RPE3 | **Total** |
| Acetobacteraceae | *Roseomonas* | - | 0 | 4 | 0 | 0 | 0 | 0 | 4 |
| Acidaminobacteraceae | Unidentified genus |  | 0 | 0 | 14 | 0 | 0 | 17 | 31 |
| Acidobacteriaceae | *Granulicella** | - | 0 | 0 | 12 | 0 | 0 | 0 | 12 |
|  | Unidentified genus |  | 0 | 0 | 0 | 0 | 19 | 0 | 19 |
| Actinomycetaceae | *Actinomyces* | + | 11 | 0 | 0 | 44 | 0 | 0 | 55 |
| Actinospicaceae | Unidentified genus |  | 0 | 0 | 2 | 0 | 0 | 0 | 2 |
| Actinosynnemataceae | *Kibdelosporangium** | - | 0 | 12 | 0 | 0 | 0 | 0 | 12 |
|  | *Kutzneria** | - | 0 | 0 | 0 | 0 | 0 | 5 | 5 |
|  | Unidentified genus |  | 0 | 0 | 0 | 0 | 0 | 8 | 8 |
| Aerococcaceae | *Facklamia* | + | 0 | 0 | 0 | 2 | 0 | 0 | 2 |
| Alcaligenaceae | *Achromobacter* | - | 7 | 6 | 0 | 0 | 0 | 0 | 13 |
|  | *Sutterella** | - | 0 | 0 | 0 | 0 | 32 | 0 | 32 |
|  | Unidentified genus |  | 4 | 0 | 0 | 0 | 0 | 0 | 4 |
| Alteromonadaceae | *Cellvibrio* | - | 21 | 0 | 0 | 0 | 0 | 6 | 27 |
| Anaplasmataceae | *Anaplasma** | - | 47 | 0 | 0 | 0 | 0 | 0 | 47 |
| Bacillaceae | *Bacillus* | + | 0 | 5 | 14 | 0 | 0 | 5 | 24 |
|  | *Oceanobacillus* | - | 0 | 0 | 0 | 18 | 12 | 7 | 37 |
|  | Unidentified genus |  | 0 | 0 | 0 | 0 | 17 | 0 | 17 |
| Bacteroidaceae | *Bacteroides** | - | 6 | 0 | 0 | 0 | 0 | 0 | 6 |
| Bartonellaceae | *Bartonella* | - | 75 | 824 | 0 | 14 | 0 | 170 | 1083 |
| Beijerinckiaceae | *Beijerinckia** | - | 0 | 0 | 6 | 0 | 0 | 0 | 6 |
| Blattabacteriaceae | Unidentified genus |  | 0 | 0 | 0 | 0 | 5 | 0 | 5 |
| Bradyrhizobiaceae | *Bosea* | - | 0 | 3 | 0 | 0 | 0 | 0 | 3 |
|  | Unidentified genus |  | 1335 | 377 | 312 | 2540 | 1505 | 914 | 6983 |
| Brevibacteriaceae | *Brevibacterium* | - | 20 | 0 | 21 | 0 | 0 | 0 | 41 |
| Burkholderiaceae | *Burkholderia* | - | 23 | 0 | 0 | 0 | 18 | 0 | 41 |
|  | *Lautropia** | + | 14 | 0 | 0 | 0 | 0 | 0 | 14 |
|  | *Salinispora** | - | 0 | 0 | 10 | 0 | 0 | 0 | 10 |
| Campylobacteraceae | *Campylobacter** | - | 29 | 36 | 26 | 7 | 0 | 23 | 121 |
| Carnobacteriaceae | *Granulicatella* | - | 0 | 0 | 0 | 13 | 0 | 0 | 13 |
| Caulobacteraceae | *Brevundimonas* | - | 2 | 0 | 12 | 17 | 0 | 0 | 31 |
|  | *Caulobacter* | - | 1203 | 263 | 267 | 511 | 1261 | 438 | 3943 |
|  | Unidentified genus |  | 43 | 11 | 0 | 0 | 0 | 0 | 54 |
| Chitinophagaceae | *Sediminibacterium** | - | 25 | 0 | 0 | 0 | 0 | 35 | 60 |
| Christensenellaceae | Unidentified genus |  | 16 | 0 | 0 | 0 | 0 | 0 | 16 |
| Clostridiaceae | Unidentified genus |  | 23 | 0 | 0 | 0 | 0 | 0 | 23 |
| Comamonadaceae | *Acidovorax* | - | 0 | 0 | 0 | 0 | 9 | 0 | 9 |
|  | *Aquabacterium* | - | 0 | 0 | 0 | 11 | 26 | 0 | 37 |
|  | *Diaphorobacter** | - | 22 | 8 | 0 | 0 | 0 | 0 | 30 |
|  | *Pelomonas* | - | 0 | 0 | 0 | 0 | 16 | 0 | 16 |
|  | Unidentified genus |  | 0 | 0 | 0 | 0 | 16 | 5 | 21 |
| Conexibacteraceae | Unidentified genus |  | 3 | 0 | 0 | 0 | 5 | 0 | 8 |
| Coriobacteriaceae | *Atopobium** | - | 605 | 659 | 428 | 417 | 131 | 38 | 2278 |
|  | *Olsenella** | - | 256 | 142 | 106 | 71 | 61 | 138 | 774 |
|  | *Slackia** | - | 0 | 33 | 5 | 0 | 0 | 40 | 78 |
|  | Unidentified genus |  | 67 | 174 | 148 | 153 | 130 | 250 | 922 |
| Corynebacteriaceae | *Corynebacterium* | + | 2 | 34 | 78 | 57 | 0 | 32 | 203 |
| Cystobacterineae | Unidentified genus |  | 0 | 0 | 53 | 0 | 0 | 0 | 53 |
| Dermabacteraceae | *Brachybacterium* | - | 7 | 5 | 0 | 38 | 0 | 0 | 50 |
| Dethiosulfovibrionaceae | Unidentified genus |  | 0 | 0 | 0 | 0 | 0 | 8 | 8 |
| Dietziaceae | *Dietzia* | - | 0 | 0 | 0 | 0 | 19 | 8 | 27 |
| Enterobacteriaceae | *Enterobacter* | - | 6 | 0 | 0 | 0 | 0 | 0 | 6 |
|  | *Rahnella* | - | 0 | 0 | 0 | 15 | 0 | 0 | 15 |
|  | *Serratia* | - | 154 | 0 | 0 | 0 | 0 | 0 | 154 |
|  | Unidentified genus |  | 46 | 43 | 0 | 0 | 16 | 16 | 121 |
| Erysipelotrichaceae | *Allobaculum** | - | 0 | 0 | 0 | 0 | 0 | 16 | 16 |
|  | *Bulleidia** | - | 19 | 47 | 0 | 0 | 0 | 0 | 66 |
|  | *Sharpea** | - | 0 | 0 | 12 | 0 | 0 | 20 | 32 |
|  | Unidentified genus |  | 0 | 13 | 0 | 0 | 0 | 8 | 21 |
| Erythrobacteraceae | *Porphyrobacter** | - | 5 | 0 | 0 | 0 | 0 | 0 | 5 |
| Frankiaceae | *Actinomycetales** | + | 0 | 0 | 0 | 2 | 0 | 0 | 2 |
|  | Unidentified genus |  | 2 | 4 | 0 | 16 | 0 | 0 | 18 |
| Fusobacteriaceae | *Fusobacterium* | - | 288 | 278 | 234 | 254 | 122 | 361 | 1537 |
|  | Unidentified genus |  | 0 | 16 | 0 | 0 | 0 | 0 | 16 |
| Gaiellaceae | Unidentified genus |  | 0 | 0 | 0 | 3 | 0 | 0 | 3 |
| Gemellaceae | Unidentified genus |  | 0 | 0 | 34 | 0 | 0 | 0 | 34 |
| Geodermatophilaceae | Unidentified genus |  | 0 | 0 | 0 | 15 | 0 | 0 | 15 |
| Halomonadaceae | *Haererehalobacter** | - | 19 | 0 | 0 | 0 | 0 | 0 | 19 |
|  | *Halomonas* | - | 17 | 0 | 0 | 0 | 0 | 0 | 17 |
|  | Unidentified genus |  | 4 | 0 | 0 | 0 | 0 | 0 | 4 |
| Holosporaceae | Unidentified genus |  | 3412 | 316 | 137 | 0 | 0 | 0 | 3865 |
| Hyphomicrobiaceae | *Hyphomicrobium* | - | 0 | 0 | 7 | 0 | 0 | 0 | 7 |
|  | *Rhodoplanes* | - | 15 | 0 | 6 | 0 | 0 | 0 | 21 |
| Intrasporangiaceae | Unidentified genus |  | 21 | 0 | 0 | 83 | 0 | 0 | 104 |
| Kineosporiaceae | Unidentified genus |  | 0 | 5 | 0 | 0 | 0 | 0 | 5 |
| Koribacteraceae | Unidentified genus |  | 0 | 8 | 0 | 13 | 0 | 0 | 21 |
| Ktedonobacteraceae | Unidentified genus |  | 0 | 3 | 0 | 0 | 0 | 0 | 3 |
| Lachnospiraceae | *Blautia** | - | 0 | 0 | 0 | 0 | 0 | 7 | 7 |
|  | *Catonella** | - | 0 | 0 | 9 | 16 | 0 | 0 | 25 |
|  | *Lachnoanaerobaculum** | - | 0 | 0 | 0 | 45 | 0 | 0 | 45 |
|  | *Moryella** | - | 48 | 5 | 34 | 32 | 29 | 11 | 159 |
|  | *Oribacterium** | - | 0 | 21 | 0 | 0 | 0 | 5 | 26 |
|  | *Shuttleworthia** | - | 0 | 5 | 11 | 0 | 0 | 9 | 25 |
|  | Unidentified genus |  | 28 | 0 | 11 | 0 | 0 | 0 | 39 |
| Lactobacillaceae | *Lactobacillus* | + | 0 | 8 | 8 | 0 | 0 | 0 | 16 |
| Leptospiraceae | *Leptospira** | - | 2 | 0 | 0 | 0 | 0 | 17 | 19 |
| Methylobacteriaceae | *Methylobacterium* | - | 0 | 0 | 0 | 0 | 6 | 0 | 6 |
| Methylocystaceae | Unidentified genus |  | 0 | 0 | 0 | 0 | 0 | 9 | 9 |
| Microbacteriaceae | *Cryocola* | - | 0 | 0 | 0 | 6274 | 0 | 0 | 6274 |
|  | *Leifsonia* | + | 0 | 0 | 0 | 31 | 0 | 0 | 31 |
|  | *Microbacterium* | + | 68 | 33 | 44 | 138 | 70 | 57 | 410 |
|  | Unidentified genus |  | 0 | 2 | 3 | 35 | 0 | 2 | 42 |
| Micrococcaceae | *Arthrobacter* | - | 0 | 7 | 0 | 0 | 2 | 0 | 9 |
|  | *Kocuria* | - | 0 | 8 | 17 | 0 | 45 | 51 | 121 |
|  | *Micrococcus* | + | 0 | 0 | 0 | 49 | 0 | 0 | 49 |
|  | *Nesterenkonia* | - | 0 | 12 | 0 | 13 | 14 | 0 | 39 |
|  | *Rothia* | - | 0 | 0 | 0 | 0 | 0 | 1606 | 1606 |
|  | Unidentified genus |  | 0 | 0 | 0 | 20 | 0 | 0 | 20 |
| Mogibacteriaceae | *Mogibacterium** | - | 37 | 24 | 18 | 18 | 0 | 24 | 121 |
|  | Unidentified genus |  | 55 | 54 | 47 | 23 | 0 | 51 | 230 |
| Moraxellaceae | *Acinetobacter* | - | 179 | 113 | 0 | 42 | 144 | 43 | 521 |
|  | *Enhydrobacter* | - | 0 | 0 | 0 | 26 | 11 | 0 | 37 |
|  | *Moraxella* | - | 0 | 0 | 0 | 15 | 0 | 0 | 15 |
|  | *Psychrobacter** | - | 0 | 0 | 20 | 0 | 0 | 0 | 20 |
|  | Unidentified genus |  | 0 | 5 | 4 | 0 | 0 | 0 | 9 |
| Mycobacteriaceae | *Mycobacterium* | - | 0 | 0 | 17 | 0 | 0 | 0 | 17 |
| Mycoplasmataceae | *Mycoplasma** | - | 2 | 49 | 16 | 26 | 0 | 67 | 160 |
| Neisseriaceae | *Aquitalea** | - | 2 | 0 | 0 | 0 | 0 | 0 | 2 |
|  | Unidentified genus |  | 0 | 0 | 0 | 32 | 0 | 0 | 32 |
| Nocardioidaceae | *Nocardioides* | - | 0 | 0 | 0 | 0 | 0 | 16 | 16 |
| Nostocaceae | Unidentified genus |  | 0 | 0 | 0 | 38 | 0 | 0 | 38 |
| Oxalobacteraceae | *Herbaspirillum* | - | 13 | 4 | 0 | 0 | 0 | 0 | 17 |
|  | *Ralstonia* | - | 15 | 9 | 0 | 0 | 12 | 0 | 36 |
|  | Unidentified genus |  | 2 | 0 | 0 | 0 | 3 | 3 | 8 |
| Paenibacillaceae | *Paenibacillus* | - | 0 | 0 | 0 | 0 | 0 | 13 | 13 |
| Pasteurellaceae | *Haemophilus* | - | 0 | 5 | 0 | 15 | 0 | 0 | 20 |
| Patulibacteraceae | *Patulibacter** | - | 7 | 0 | 0 | 0 | 0 | 0 | 7 |
|  | Unidentified genus |  | 0 | 0 | 0 | 12 | 0 | 0 | 12 |
| Peptococcaceae | *Peptococcus** | + | 0 | 0 | 4 | 33 | 5 | 4 | 46 |
| Peptostreptococcaceae | *Filifactor** | - | 222 | 142 | 139 | 175 | 21 | 102 | 801 |
|  | *Peptostreptococcus* | + | 697 | 675 | 678 | 639 | 239 | 484 | 3412 |
|  | Unidentified genus |  | 35 | 0 | 3 | 39 | 0 | 0 | 74 |
| Planococcaceae | *Staphylococcus* | + | 18 | 31 | 0 | 50 | 0 | 184 | 283 |
| Porphyromonadaceae | *Porphyromonas** | - | 1457 | 1973 | 1257 | 1357 | 184 | 1420 | 7648 |
|  | *Tannerella** | - | 0 | 10 | 0 | 9 | 16 | 0 | 35 |
|  | Unidentified genus |  | 0 | 0 | 0 | 0 | 0 | 6 | 6 |
| Prevotellaceae | *Prevotella* | - | 3318 | 3088 | 2363 | 2392 | 863 | 1833 | 13857 |
|  | Unidentified genus |  | 4 | 10 | 0 | 0 | 0 | 4 | 18 |
| Propionibacteriaceae | *Propionibacterium* | + | 5 | 0 | 6 | 127 | 24 | 12 | 174 |
|  | Unidentified genus |  | 0 | 0 | 0 | 0 | 0 | 41 | 41 |
| Pseudomonadaceae | *Pseudomonas* | - | 185 | 126 | 0 | 94 | 0 | 0 | 405 |
|  | Unidentified genus |  | 48 | 6 | 0 | 38 | 0 | 4 | 90 |
| Pseudonocardiaceae | *Actinomycetospora** | + | 0 | 0 | 0 | 0 | 0 | 5 | 5 |
|  | *Pseudonocardia* | - | 0 | 0 | 0 | 0 | 10 | 0 | 10 |
|  | Unidentified genus |  | 0 | 9 | 0 | 0 | 0 | 0 | 9 |
| Rhizobiaceae | *Agrobacterium* | - | 3 | 0 | 0 | 0 | 0 | 0 | 3 |
|  | *Rhizobium* | - | 0 | 0 | 0 | 0 | 0 | 6 | 6 |
| Rhodospirillaceae | *Reyranella* | - | 55 | 8 | 43 | 54 | 30 | 47 | 237 |
|  | Unidentified genus |  | 11 | 0 | 0 | 0 | 0 | 0 | 11 |
| Rickettsiaceae | *Rickettsia* | - | 59044 | 52793 | 40527 | 23494 | 41983 | 60703 | 278544 |
|  | Unidentified genus |  | 0 | 0 | 7 | 0 | 0 | 15 | 21 |
| Ruminococcaceae | *Faecalibacterium** | - | 0 | 3 | 0 | 0 | 0 | 0 | 3 |
| Simkaniaceae | *Fritschea** | - | 0 | 0 | 27 | 4 | 0 | 0 | 31 |
| Sinobacteraceae | *Nevskia** | - | 10 | 7 | 10 | 0 | 0 | 0 | 27 |
|  | Unidentified genus |  | 995 | 162 | 1185 | 1653 | 2530 | 648 | 7173 |
| Sphingobacteriaceae | Unidentified genus |  | 0 | 0 | 0 | 0 | 10 | 0 | 10 |
| Sphingomonadaceae | *Novosphingobium* | - | 48 | 0 | 0 | 0 | 49 | 0 | 97 |
|  | *Sphingomonas* | - | 124 | 4 | 89 | 141 | 202 | 86 | 646 |
|  | *Sphingopyxis* | - | 13 | 0 | 0 | 0 | 0 | 0 | 13 |
|  | Unidentified genus |  | 0 | 0 | 0 | 2 | 0 | 0 | 2 |
| Spirochaetaceae | *Treponema** | - | 8 | 0 | 0 | 0 | 0 | 9 | 17 |
| Staphylococcaceae | *Staphylococcus* | + | 18 | 31 | 0 | 50 | 0 | 184 | 283 |
| Streptococcaceae | *Streptococcus* | + | 34 | 25 | 19 | 92 | 6 | 0 | 176 |
| Syntrophobacteraceae | Unidentified genus |  | 0 | 0 | 0 | 0 | 13 | 0 | 13 |
| Thermogemmatisporaceae | Unidentified genus |  | 0 | 0 | 0 | 30 | 0 | 0 | 30 |
| Tissierellaceae | *Parvimonas** | - | 169 | 98 | 115 | 176 | 40 | 98 | 696 |
|  | *Peptoniphilus* | + | 0 | 0 | 0 | 34 | 0 | 0 | 34 |
|  | Unidentified genus |  | 9 | 21 | 3 | 0 | 0 | 0 | 33 |
| Veillonellaceae | *Dialister** | - | 28 | 56 | 76 | 28 | 0 | 49 | 237 |
|  | *Selenomonas* | - | 19 | 10 | 10 | 0 | 0 | 17 | 56 |
|  | *Veillonella* | - | 0 | 0 | 0 | 15 | 9 | 0 | 24 |
|  | Unidentified genus |  | 0 | 0 | 10 | 0 | 0 | 2 | 12 |
| Weeksellaceae | *Chryseobacterium* | - | 0 | 0 | 0 | 0 | 12 | 0 | 12 |
|  | *Wautersiella* | - | 42 | 95 | 0 | 112 | 5 | 3 | 257 |
| Xanthomonadaceae | *Dyella** | - | 0 | 0 | 14 | 0 | 0 | 0 | 14 |
|  | *Silanimonas** | - | 11 | 0 | 5 | 0 | 0 | 0 | 16 |
|  | *Stenotrophomonas* | - | 9 | 0 | 0 | 0 | 0 | 0 | 9 |
| Xenococcaceae | Unidentified genus |  | 0 | 0 | 0 | 0 | 0 | 2 | 2 |
|  | **Total** |  | 74971 | 63076 | 48793 | 42052 | 50007 | 70527 | 349412 |

*Bacterial genera recorded for the first time in sandflies.
